# Dapagliflozin as an adjunct therapy to insulin in the treatment of patients with type 1 diabetes mellitus

**DOI:** 10.1186/s40200-015-0210-x

**Published:** 2015-10-09

**Authors:** Hector E. Tamez, Alejandra L. Tamez, Lucas A. Garza, Mayra I. Hernandez, Ana C. Polanco

**Affiliations:** Subdirección de Investigación, Facultad de Medicina y Hospital Universitario Dr. José Eleuterio González, Calle Aguirre Pequeño, S/N, Monetrrey, N.L. 64460 México

**Keywords:** Type 1 diabetes mellitus, Dapagliflozin, Insulin

## Abstract

We have evaluated the efficacy of dapagliflozin in patients with type 1 diabetes mellitus (DM1) without adequate control. We expected that adding dapagliflozin to this population on top of their base treatment would lower their HbA1c levels.

We conducted a pragmatic, open, 24-week study of treatment with 10 mg of oral dapagliflozin in patients with DM1 and chronic hyperglycemia. We evaluated glycemic control, lipid profile, weight, and insulin dose. Safety was assessed by adverse event reporting.

Fasting glucose levels decreased from 176.42 ± 45.33 mg/dL to 139.67 ± 44.42 mg/dL (p = 0.05); although no significant valued was reached, postprandial glucose showed a decreased tendency from 230.25 ± 52.06 mg/dL to 193.83 ± 45.43 mg/dL (*p* = 0.08). The hemoglobin A1C (HbA1C) level decreased from 9.18 ± 1.02 (77 ± 11.1 mmol/mol) to 8.05 ± 1.09 % (64 ± 11.9 mmol/mol) (*p* = 0.0156); total cholesterol decreased from 299 ± 12 to 199 ± 7 mg/dL (*p* = 0.02); triglycerides decreased from 184 ± 15 to 160 ± 11 mg/dL (*p* = 0.0002), HDL-C decreased from 40 ± 17 to 42 ± 9 mg/dL (*p* = 0.54); and LDL-C decreased from 187 ± 19 to 170 ± 21 mg/dL (*p* = 0.049). No adverse events were reported.

The beneficial effects of SGLT2 inhibitors on metabolic control and their safety after a 24-week open study demonstrate their potential indication as an adjunctive treatment with insulin in patients with DM1; however, long-term clinical trials should be considered.

Treatment of Type 1 diabetes mellitus (DM1) is presently restricted to insulin and in selected cases, pramlintide and islet or pancreas transplantation [1–3].

In contrast the management of DM1 with oral drugs is limited. Sodium/glucose cotransporter 2 inhibitors (SGLT2I), as initial or in combination therapy have recently been used to treat type 2 diabetes mellitus (DM2) [4]. These drugs improve glycemic control, promote weight loss and lower arterial tension; meanwhile, SGLT-2 studies in DM1 are scarce or short-timed [5–8].

The aim of this study was to evaluate the efficacy of dapagliflozin in a group of patients with poorly controlled DM1. We expected that adding dapagliflozin to this population’s insulin regimen, would improve their glycemic control.

## Research design and methods

We performed an open, 24-week, pragmatic clinical trial in a group of patients with poorly controlled DM1 in a private clinic in Monterrey, Nuevo Leon, México from 2013–2014. Twelve patients met the following inclusion criteria: DM1 according to the American Diabetes Association classification [9]; both genders; age greater than 18 years; and elevated glycated hemoglobin A1C (HbA1C) DDCCT Units (7–10 %) and IFCC Units (53–85.8 mmol/mol). Patients that presented micro- and macrovascular, acute hyperglycemia <300 mg/gl (16.64 mmol/L), ketonuria, ketoacidosis or hyperosmolarity in the last 6 months or pregnancy were excluded. Oral dapagliflozin 10 mg was administered daily to the entire group. Their original insulin regimen (basal/bolus) remained unchanged. Patients were instructed on detecting hypoglycemia symptoms and checking their blood glucose.

Changes in fasting and postprandial glucose (2 h after the meal) and HbA1C were considered to be the primary outcomes. Appearance of hypoglycemia, genitourinary or micotic infections and ketonuria were routinely revised.

The study protocol was approved by the local research ethics committee.

## Results

Twelve patients were included (seven male, five female) with a mean age of 27 ± 11 years and a mean duration of disease of 9.17 ± 7.41 years. All were overweight with a body mass index (BMI) of 27.98 ± 1.97 kg/m^2^.

The fasting glucose decreased from 176.42 ± 45.33 mg/dL to 139.67 ± 44.42 mg/dL (*p* = 0.05). The postprandial glucose decreased from 230.25 ± 52.06 mg/dL to 193.83 ± 45.43 mg/dL; however, it did not reach a significant value (*p* = 0.08). HbA1C significally decreased from 9.18 ± 1.02 % (77 ± 11.1 mmol/mol) to 8.05 ± 1.09 % (64 ± 11.9 mmol/mol) (*p* = 0.0156). The lipid profile was modified favorably, with total cholesterol decreasing from 299 ± 12 to 199 ± 7 mg/dL (*p* = 0.02), LDL-C from 187 ± 19 to 170 ± 21 mg/dL (*p* = 0.049) and triglycerides from 184 ± 15 to 160 ± 11 mg/dL (*p* = 0.0002). HDL-C was unchanged at 40 ± 17 and 42 ± 9 mg/dL (*p* = 0.54) (Table 1).

**Table 1:** Comparison of efficacy

Variables (*N* = 12)	Initial	Final	*P* value
Sex (M/male,F/female)	M: 7 / F:5		
Age (years)	27.67 ± 14		
Evolution of DM1 (years)	9.17 ± 7.41		
Weight (kg)	78.33 ± 12.06	76.83 ± 11.72	0.76
Insulin U/kg	0.98 ± 0.11	0.96 ± 0.11	0.44
FG (mg/dl)^a^	176.42 ± 45.33	139.67 ± 44.42	0.05
PPG (mg/dl)^b^	230.25 ± 52.06	193.83 ± 45.43	0.08
HbA1C (%)	9.18 ± 1.02	8.05 ± 1.09	0.0156
HbA1C (mmol/mol)^c^	77 ± 11.1	64 ± 11.9	0.0156
Total Cholesterol (mg/dL)	299 ± 12	199 ± 7	0.02
HDL-C (mg/dL)	40 ± 7	42 ± 9	0.54
LDL-C (mg/dl)	187 ± 19	170 ± 21	0.049
Triglycerides (mg/dL)	184 ± 15	160 ± 11	0.0002

Values are means ± SD

^a^FG: Fasting glucose

^b^PPG: postprandial glucose

^c^According to the Harmonizing Hemoglobin A1c testing NGSP

Dapagliflozin was well tolerated. Hypoglycemia, infections, ketonuria or ketoacidosis were not seen.

## Discussion

The current report describes a medium-term interventional study in DM1 patients treated daily with 10 mg dapagliflozin. Treatment with dapagliflozin resulted in an improvement of both fasting and postprandial glucose and HbA1C, which may have resulted from a longer treatment period than in previous studies (up to 8 weeks) [8].

The lipid profile also improved following treatment, with reductions in total cholesterol, c-LDL cholesterol and triglycerides (all statistically significant). This is inconsistent with previous SGLT-2 studies and may have resulted from improved glycemic control, weight loss, or other causes [4, 9].

An unattained metabolic control of DM1 has prompted a search for other therapeutic options, like pramlintide or islets transplantation [1–3, 10–12].

Evidence of efficacy of SGLT2I in DM1 is limited [13]; however, studies are beginning to appear. Use of remogliflozin has been stated in small reports, along with basic studies of SGLT-2I in rats [5, 6, 14]. A case series in individuals with DM1 reported favorable results in reducing HbA1C, weight, insulin dose, and hypoglycemic events; furthermore, it appears SGLT-2I could decrease the renal hyperfiltration in DM1 patients [5–8]. Another study that included 40 normotensive patients over a period of 8 weeks reported the pleiotropic actions of SGLT2I and improvements in cardiovascular risk [8]. A recent report of dapagliflozin study in adults with type 1 diabetes demonstrated acceptable short-term tolerability, and expected pharmacokinetic profiles and increases in urinary glucose excretion [15].

We believe that dapagliflozin is safe and well tolerated with no clinically documented adverse effects. In addition, our study had the strength of a pragmatic design with a relatively long treatment period.

However, recently the U.S. Food and Drug Administration (FDA) issued a Drug Safety Communication warning of an increased risk of diabetic ketoacidosis associated with the used of all the approved SGLT2I. This potential complication related is predictable, detectable, and preventable, with the full picture still favoring the use of SGLT2I DM1 patient [16].

Nevertheless, we recognize that the data should be cautiously considered because the study was performed in a specialized care center with educated patients and a small sample size of overweight individuals. Also, we do not have information regarding the follow up of the patients.

## Conclusions

Dapagliflozin as an adjunct therapy to insulin caused significant changes in the HbA1C level, fasting and postprandial glucose and atherogenic lipid in patients with DM1. Thus, dapagliflozin may represent a new therapeutic approach, although long-term controlled clinical trials with a greater number of patients are needed to confirm our data.
